# Supplementary material for: Comprehensive multi-omics analysis of CD36 in pan-cancers: Evaluating role in prognosis, immune microenvironment, and therapeutic response
Source: Pharm Sci Adv. 2025 Sep 30;3:100097. doi: 10.1016/j.pscia.2025.100097 (PMC12709870; doi:10.1016/j.pscia.2025.100097)
Supplement: Multimedia component 1 [file mmc1.docx]

**
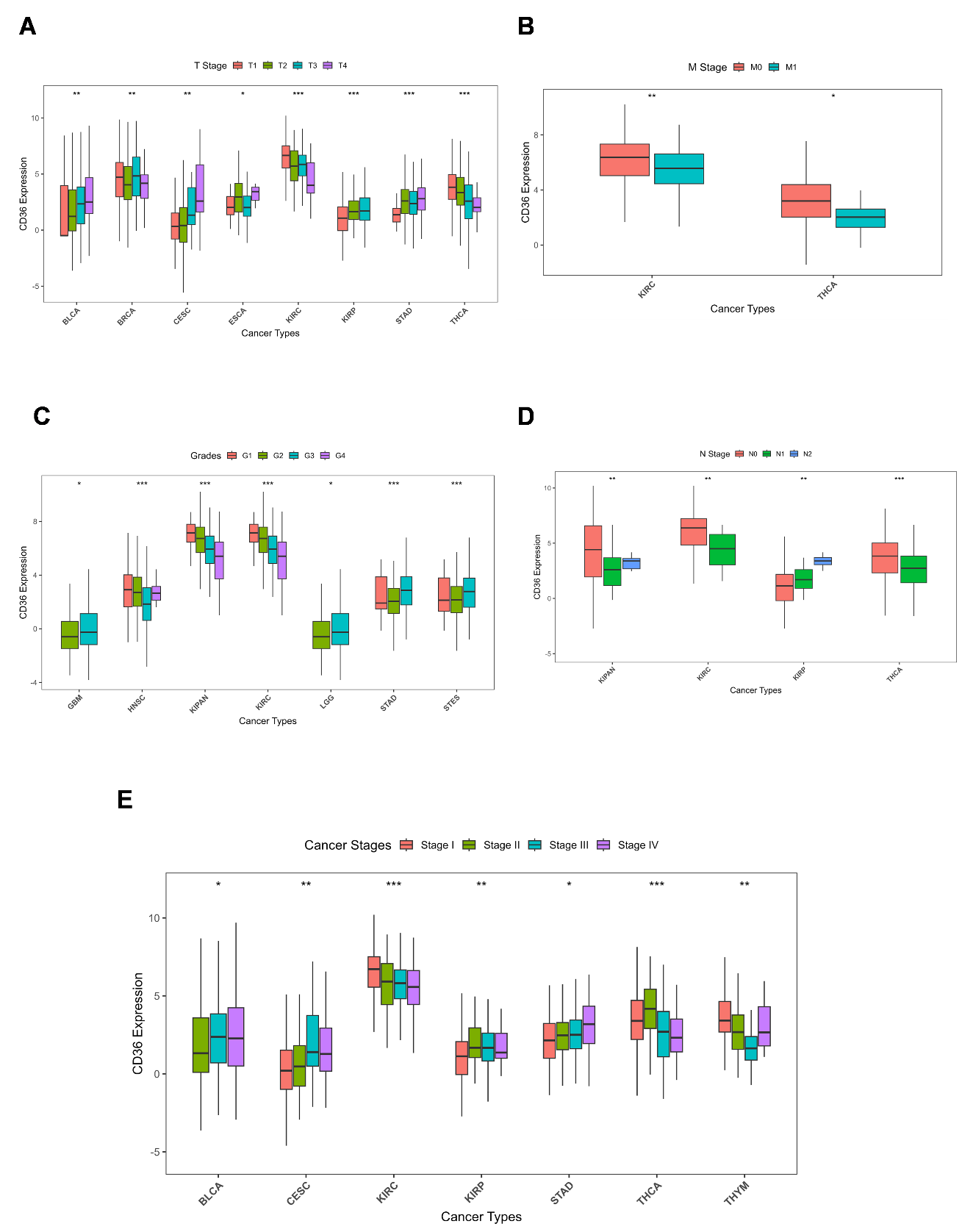
**

**Fig. S1 CD36 association with pan-cancer clinical characteristics.** (**A**) Tumor (T), (**B**) Metastasis (M), (**C**) Grades, (**D**) Nodes (N), and (**E**) Cancer Stages **P* < 0.05, ***P* < 0.01, ****P* < 0.001.

**
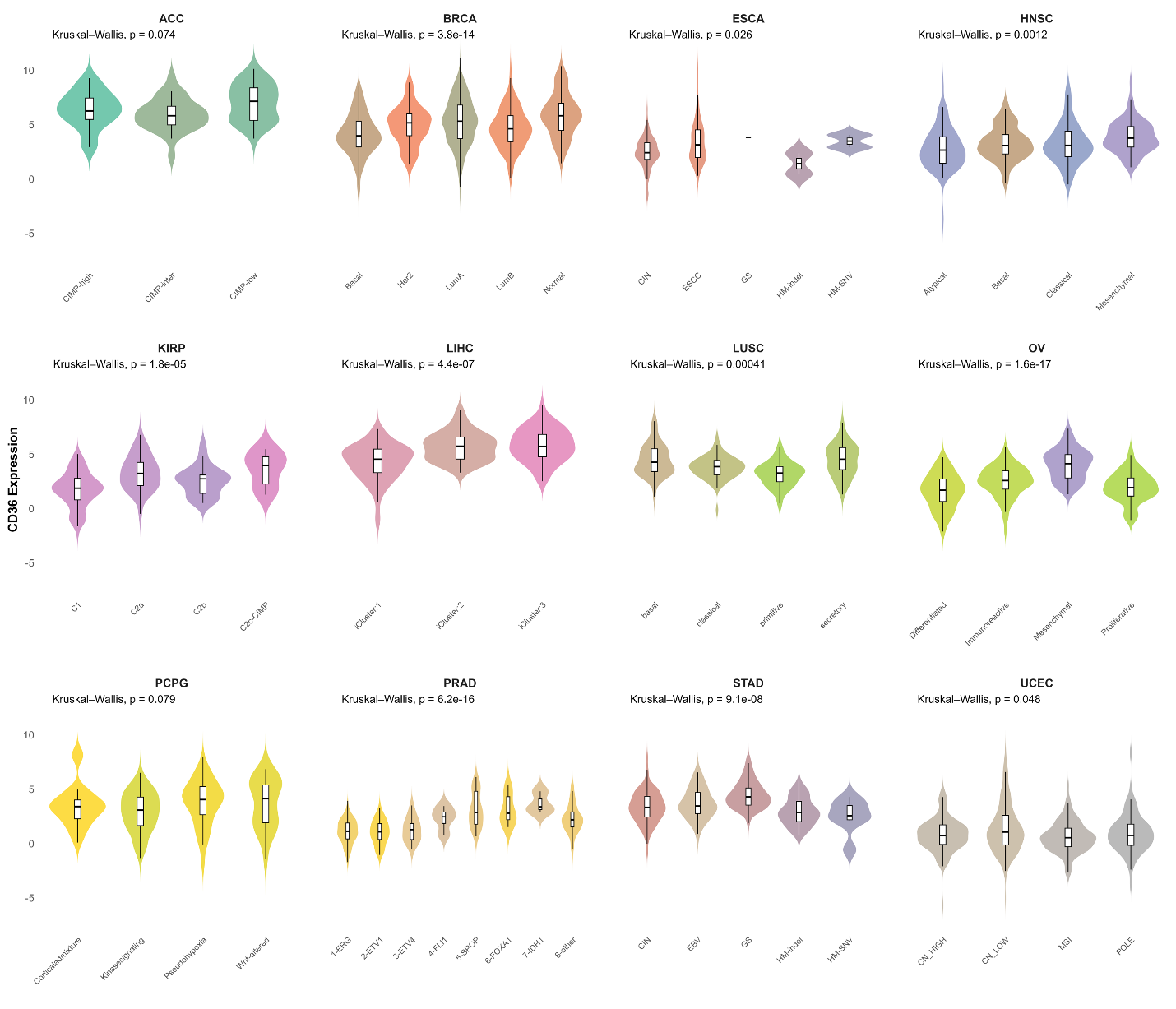
**

**Fig. S2 CD36 expression in molecular subtypes of various cancer types**

**
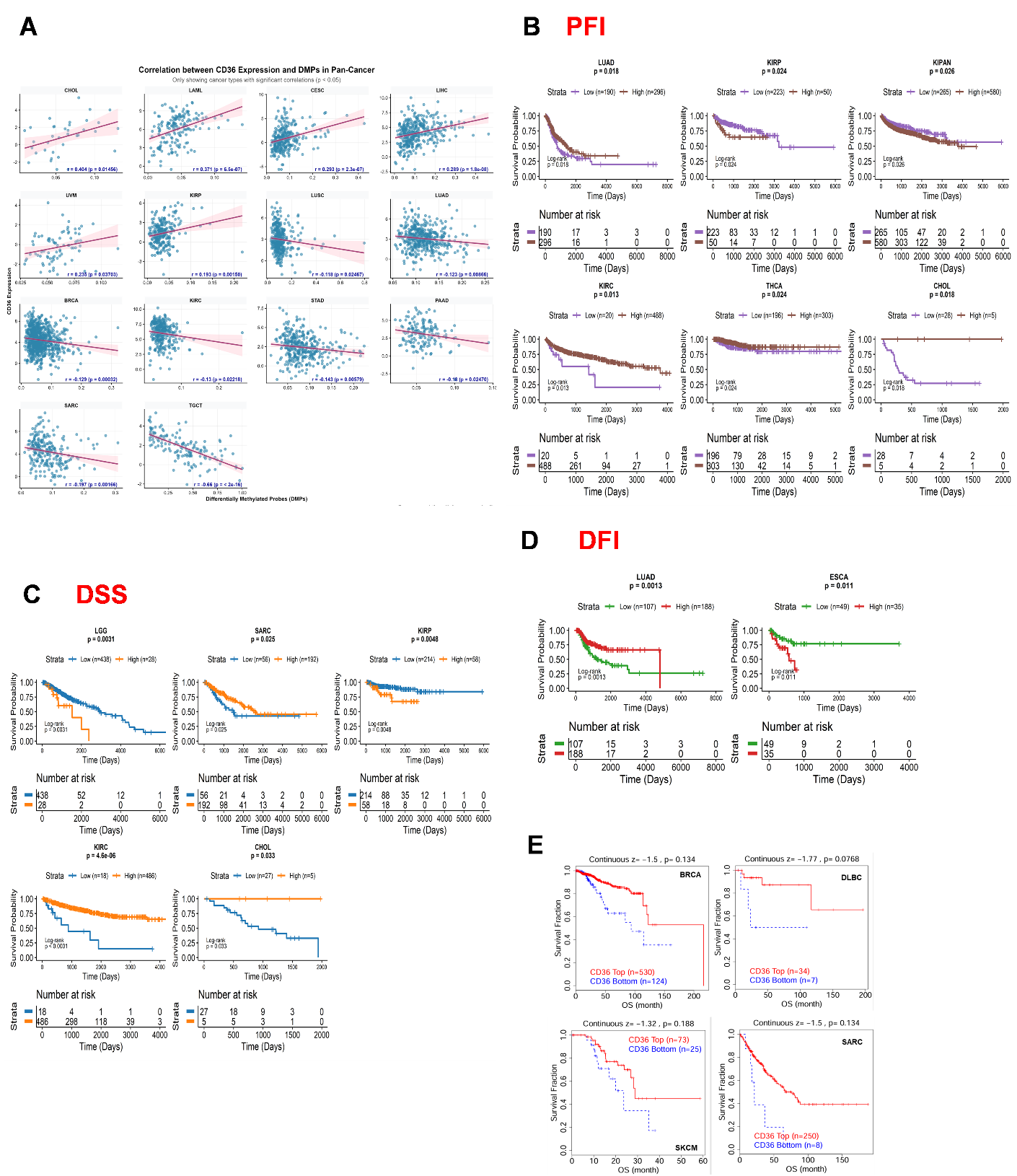
**

**Fig. S3 Prognostic significance and epigenetic regulation of CD36 in pan-cancer.** (**A**) Correlation between CD36 expression and DMPs in pan-cancer; (**B**) KM curve to evaluate the association between CD36 and progression free interval (PFI) in pan-cancer; (**C**) KM curve to evaluate the association between CD36 and disease specific survival (DSS) in pan-cancer; (**D**) KM curve to evaluate the association between CD36 and disease free survival (DFI) in pan-cancer; (**E**) Correlation between CD36 methylation level and clinical prognosis of BRCA, SKCM, DLBC and SARC based on the TIDE database.

**
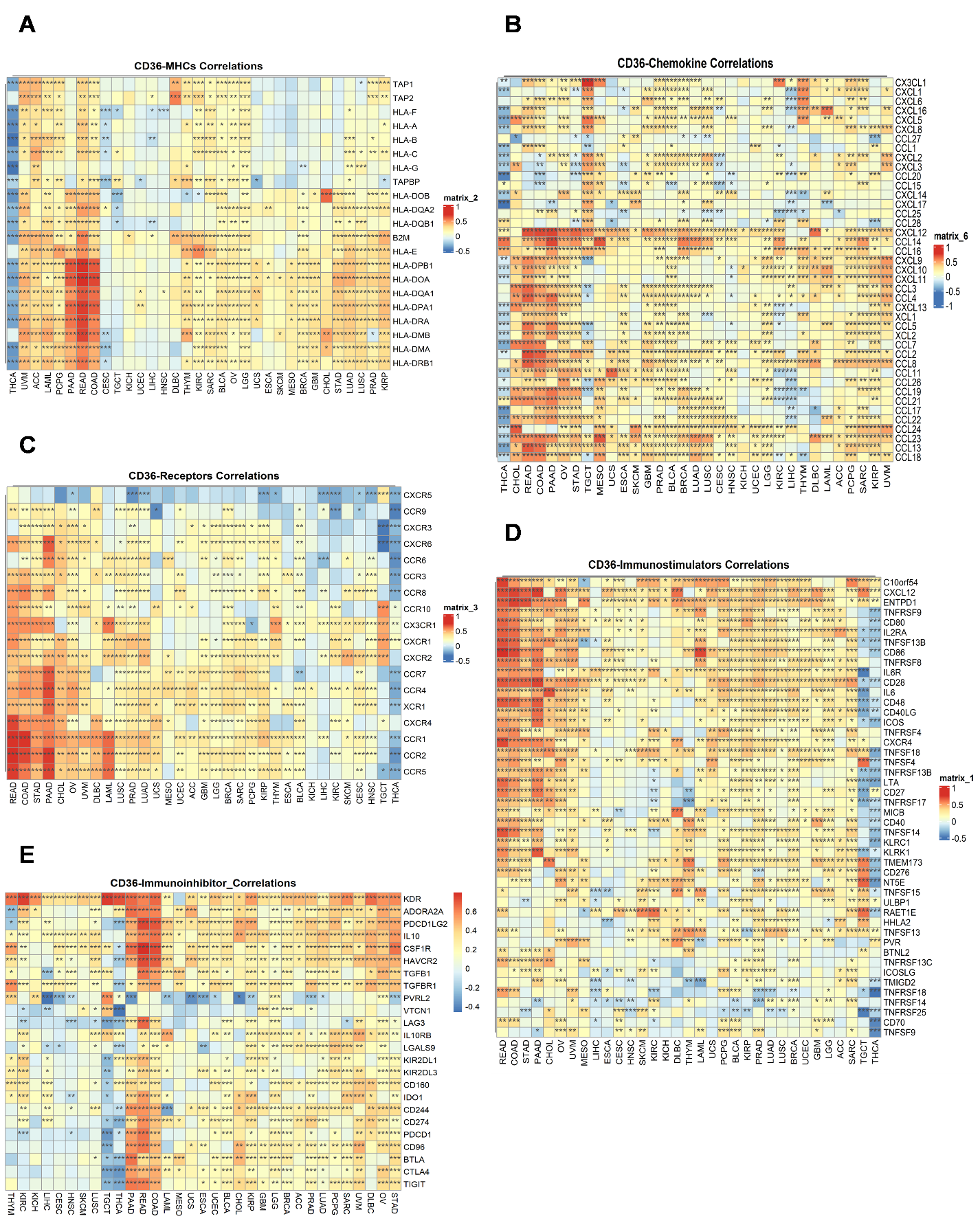
**

**Fig. S4 Correlation between CD36 and different immune regulators** including (**A**) MHC, (**B**) chemokines, (**C**) receptors, (**D**) immunostimulators, and (**E**) immunoinhibitors **P* < 0.05, ***P* < 0.01, ****P* < 0.001.
